# Chromosome-scale genome assemblies of *Himalopsyche anomala* and *Eubasilissa splendida* (Insecta: Trichoptera)

**DOI:** 10.1038/s41597-024-03097-3

**Published:** 2024-03-05

**Authors:** Xinyu Ge, Lang Peng, Zhen Deng, Jie Du, Changhai Sun, Beixin Wang

**Affiliations:** 1 Department of Entomology, College of Plant Protection, Nanjing Agricultural University, Nanjing, 210095 China; 2 Tianjin Key Laboratory of Conservation and Utilization of Animal Diversity, College of Life Sciences, Tianjin Normal University, Tianjin, 300387 China; 3 Jiuzhaigou Administration Bureau, Jiuzhaigou County, Aba Prefecture, Sichuan Province 623402 China

**Keywords:** Entomology, Comparative genomics

## Abstract

Trichoptera is one of the most evolutionarily successful aquatic insect lineages and is highly valued value in adaptive evolution research. This study presents the chromosome-level genome assemblies of *Himalopsyche anomala* and *Eubasilissa splendida* achieved using PacBio, Illumina, and Hi-C sequencing. For *H. anomala* and *E. splendida*, assembly sizes were 663.43 and 859.28 Mb, with scaffold N50 lengths of 28.44 and 31.17 Mb, respectively. In *H. anomala* and *E. splendida*, we anchored 24 and 29 pseudochromosomes, and identified 11,469 and 10,554 protein-coding genes, respectively. The high-quality genomes of *H. anomala* and *E. splendida* provide critical genomic resources for understanding the evolution and ecology of Trichoptera and performing comparative genomics analyses.

## Background & Summary

Trichoptera, commonly known as caddisflies, represent the largest order of completely aquatic insects within Endopterygota^1^. Encompassing approximately 17,000 extant species, Trichoptera are distributed across all continents except Antarctica^2^. Their larvae exhibit remarkably diverse behavior, constructing various nest structures or living freely in aquatic environments^3^. Their adaptability to varying water conditions, including temperature and dissolved oxygen, differs significantly among families, genera, and individual species^4^. Consequently, they serve as vital indicator organisms in water quality monitoring efforts. Additionally, the varied feeding habits of trichopteran larvae contribute to the energy dynamics within stream ecosystems^5,6^.

Trichoptera is divided into two suborders, Annulipalpia and Integripalpia, based on morphology and habit. Annulipalpian larvae typically inhabit running water or wave-washed riverbanks, using pin silk along with plant debris and small stones to construct fixed shelter. Integripalpia includes “cocoon-makers” and “Phryganides”^7,8^. Cocoon-makers larvae are either free-living or construct purse-case or saddle-case and are usually found in fast-flowing rivers and streams. Last instar larvae produce closed, semipermeable cocoons for pupation. In contrast, most Phryganides larvae thrive in stagnant or slow-moving water, adeptly combining stones, leaves, and twigs with silk proteins to construct mobile nests^9,10^. Rhyacophilidae and Phryganeidae are representative cocoon-makers and Phryganides, respectively, and exhibit marked ecological habit and lifestyle differences.

The family Rhyacophilidae originated in the Palaearctic region and is primarily distributed in the northern-hemisphere^11^. Their predatory larvae exhibit high sensitivity to environmental changes^12^. However, the majority of phryganeid larvae are shredders, feeding on detritus and plant material in aquatic environments^13^. These larvae tend to be less sensitive to environmental changes compared with rhyacophilid larvae. Some species can survive in humid terrestrial environments after leaving the water^10^. *Himalopsyche anomala* Banks and *Eubasilissa splendida* Yang & Yang are typical representatives of Rhyacophilidae and Phryganeidae, respectively. Despite extensive studies on their biological characteristics, their precise phylogenetic positions and the molecular mechanisms underlying their adaptive evolution remain uncertain. High-quality reference genomes are crucial for advancing genetics and genome research. To date, nearly 30 trichopteran species have had their genomes sequenced and published, including two *Himalopsyche* species and *Eubasilissa regina*. However, the chromosome-level has been reached in only partial species from five families (Glossosomatidae, Hydropsychidae, Leptoceridae, Limnephilidae, and Odontoceridae).

To enhance our understanding of the adaptive evolution and ecology of holometabola aquatic insects, we used PacBio long-read sequencing, Illumina short-read sequencing, and Hi-C data sequencing techniques to achieve the first chromosome-level genome assemblies for *H. anomala* Banks and *E. splendida* Yang & Yang, with assembly sizes of 663.43 and 859.28 Mb and scaffold N50 lengths of 28.44 and 31.17 Mb, respectively. Hi-C scaffolding resulted in chromosome-level assemblies, with 99.29% (2,697 contigs) and 99.61% (643 contigs) of the initially assembled sequences anchored to 24 and 29 pseudochromosomes for *H. anomala* and *E. splendida*, respectively. In total, 288.10 Mb (43.43%) and 471.23 Mb (54.84%) of the sequences were identified as repetitive elements in these two respective assemblies. Moreover, integrating three prediction methods enabled the identification of 11,469 and 10,554 protein-coding genes (PCGs) in *H. anomala* and *E. splendida*, respectively. The high-quality genomes of these species not only advance our understanding of adaptive evolution in Trichoptera but also serve as resources for comparative genomics research on evolution in biology and ecology fields. Furthermore, they contribute to elucidating the phylogenetic relationships between the cocoon-maker and Phryganides groups.

## Methods

### Sample collection

*Himalopsyche anomala* and *E. splendida* specimens were collected using ultraviolet light tubes from Xi-niu Sea (33°11′42″N; 103°53′46″E; alt: 2,348 m) and Wu-hua Sea (33°09′32″N; 103°51′55″E; alt: 2,377 m), respectively, in Jiuzhaigou National Nature Reserve, Sichuan Province, in July 2020. Specimens were identified by X-Y Ge and C-H Sun. Each sample underwent cleaning with phosphate-buffered saline buffer and the gut was removed under a stereo microscope (to minimize intestinal microbial contamination). Subsequently, samples were stored in liquid nitrogen before nucleic acid extraction^14^.

### Nucleic acid extraction and sequencing

For genome survey, transcriptome, PacBio, and Hi-C sequencing, four male individuals of each species were sequenced. Additionally, a female individual underwent DNA sequencing using the Illumina platform to identify sex chromosome. DNA and RNA were extracted from samples using the Qiagen DNeasy Blood & Tissue Kit (Qiagen) and TRIzol Reagent Kit (Invitrogen)^15^.

For PacBio sequencing, sequencing libraries with 20 kb (*H. anomala*) and 30 kb (*E. splendida*) insert size were constructed, respectively, using the SMRTbell Template Prep Kit 1.0-SPv3, tailored to the quality of extracted DNA. Long-read sequencing was performed using the PacBio Sequel II platform with the CLR strategy. PCR-free sequencing libraries with a 350 bp insert size were generated for short-read genome sequencing. The Hi-C library was created using Mbol restriction endonuclease^16^. Both library types were subsequently sequenced on the Illumina Novaseq. 6000 and BGISEQ-500 platforms.

In total, approximately 285.76 and 352.18 Gb of raw data were generated for *H. anomala* and *E. splendida*, respectively. For *H. anomala*, the raw data included 117.23 Gb (approximately 176×) of PacBio reads with a scaffold N50 of 19.78 kb, 86.45 Gb of Illumina reads (comprising 28.87 and 57.58 Gb from the female and male samples, respectively), 74.62 Gb of Hi-C data, and 6.11 Gb of transcriptome data. For *E. splendida*, the raw data consisted of 117.9 Gb (approximately 136×) of PacBio reads with a scaffold N50 of 29.33 kb, 131.42 Gb of Illumina reads (comprising 43.73 and 87.69 Gb from the female and male samples, respectively), 91.40 Gb of Hi-C data, and 6.16 Gb of transcriptome data.

### Genome size estimation and assembly

The acquired DNA sequencing reads underwent rigorous quality control using BBmap v38.67^17^. This process included the removal of duplicate reads and filtering of low-quality reads, which were defined as follows: quality score < 20, length < 15, and consecutive polymer A/G/C > 10. For k-mer analysis, khist.sh was used with the parameter k = 21. Genome size was estimated using the R package of GenomeScope v2.0.1^18^ to calculate the k-mer distribution and generate a histogram, with a maximum sequencing coverage of 10,000. The estimated genome sizes were approximately 608.17 and 786.73 Mb for *H. anomala* and *E. splendida*, respectively, with the *H. anomala* genome exhibiting higher heterozygosity (1.03%; Fig. S1) compared to the lower heterozygosity of *E. splendida* (0.79%; Fig. S2).

**Fig. 1:**
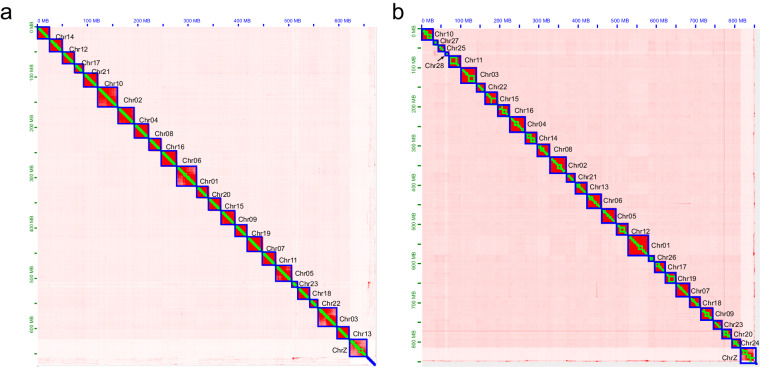
Genome-wide chromosomal interactive heatmap. Each chromosome and contig is framed in blue and green, respectively. (**a**) *Himalopsyche anomala*. (**b**) *Eubasilissa splendida*.

Flye v2.8.3^19^ was used for PacBio long-read assembly, with one round of self-polishing based on long reads. This resulted in 774.15 and 870.01 Mb assemblies for *H. anomala* and *E. splendida*, respectively. Illumina short-read mapping was performed using Minimap2 v2.17^20^, and the assembled genome underwent two rounds of polishing with NextPolish v1.1.0^21^. Redundant sequences were removed using Purge_Dups v1.2.5^22^ with the haploid cutoff set at 60 (-s 60) based on the aforementioned short-read mapping. Before chromosome anchoring, Hi-C reads alignment and quality control were conducted using Juicer v1.6.2^23^ with its default parameters. Subsequently, 3D-DNA v180922^24^ was employed to automatically anchor the majority of contigs into pseudochromosomes. Mis-joins were corrected using Juicebox v1.11.08^23^ through manual inspection and refinement. In total, 97.68% and 99.58% of assembly contigs were anchored into 24 and 29 pseudochromosomes, with lengths of 11.53–39.79 Mb for *H. anomala* and 9.92–51.78 Mb for *E. splendida* (Fig. 1).

Thorough examination for potential contaminants was conducted using MMseqs. 2 v11^25^ with the parameter “–min-seq-id 0.8” against the National Center for Biotechnology Information (NCBI) nt and UniVec databases. Sequences with > 90% alignments were removed. The final assembly lengths were 663.43 Mb (*H. anomala*) and 859.28 Mb (*E. splendida*), respectively (Table 1). To identify sex chromosomes, Illumina reads of the female individual were mapped against the assembly, and sequencing depth for each chromosome was calculated. Trichoptera follows the ZO female sex determination system^26^, hence, chromosomes with half the sequencing depth were identified as sex chromosomes (Tables S1, S2). The GC content of *H. anomala* and *E. splendida* assemblies was 31.55% and 32.76%, respectively. Notably, the estimated genome size closely matched the assembly size, with the genome assembly size of *H. anomala* resembling that of other *Himalopsyche* species^27,28^, whereas the genome size of *E. splendida* exceeded that of *Eubasilissa regina* (440.07 Mb)^29^. Genome completeness was assessed using Benchmarking Universal Single-Copy Orthologs (BUSCO) v3.0.2^30^, employing the parameter “-m genome”, during each stage of the assembly. The completeness was computed as 98.1% and 98.2% for *H. anomala* and *E. splendida*, respectively, indicating high-quality assembled genomes (Table 2).

**Table 1:** Genome assembly statistics for *Himalopsyche anomala* and *Eubasilissa splendida*.

Assembly	*Himalopsyche anomala*	*Eubasilissa splendida*
Total contig/scaffold Number	3,084/402	935/321
Total Length (MB)	663.433	859.28
contig/scaffold N50 (MB)	0.48/23.32	4.68/22.87
Max contig length: (MB)	39.791	51.78
Max scaffold Length (MB)	6.762	17.607
Gap (%)	0.04	0.01
GC Content (%)	31.55	32.76

**Table 2:** Statistical result of BUSCO for *Himalopsyche anomala* and *Eubasilissa splendida*.

Summary	*Himalopsyche anomala*	*Eubasilissa splendida*
Complete BUSCOs	1,342 (98.1%)	1,343 (98.2%)
Complete and single-copy BUSCOs	1,328 (97.1%)	1,336 (97.7%)
Complete and duplicated BUSCOs	14 (1.0%)	7 (0.5%)
Fragmented BUSCOs	6 (0.4%)	5 (0.4%)
Missing BUSCOs	19 (1.5%)	19 (1.4%)

### Repetitive sequence and noncoding RNAs annotation

RepeatModeler v2.0.2^31^ and the LTR discovery pipeline (-LTRstruct) of genome tools^32^ were used to build a *de novo* repetitive element database. Subsequently, we merged this database with the known repeat element database (Repbase-20181026^33^ and Dfam 3.1^34^). RepeatMasker v4.0.7^35^ was used to annotate the repeat elements of the two assemblies based on the custom database, identifying 288.10 Mb (approximately 43.43%) and 471.23 Mb (approximately 54.84%) of repetitive sequences for *H. anomala* and *E. splendida*, respectively. Among these elements, the largest proportion comprised unclassified elements, accounting for 21.43% and 28.44% of the total genomes of the respective species. Details regarding other common repetitive elements are provided in Tables S3, S4. To annotate the non-coding RNAs, we employed Infernal v1.1.4^36^ and tRNAscan-SE v2.0.9^37^, low-confidence tRNAs by setting parameter “EukHighConfidenceFilter” was filtered. A total of 717 ncRNAs and 766 ncRNAs were annotated in the *H. anomala* and *E. splendida* genomes, respectively, with tRNAs constituting more than 50% (384 and 420) of these ncRNAs. Details regarding other noncoding RNAs are provided in Tables S5, S6.

### Genome annotation

**Fig. 2:**
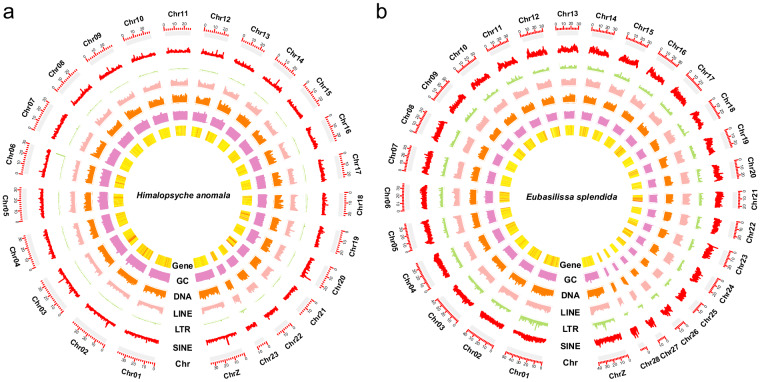
Characterization of the assembled *Himalopsyche anomala* and *Eubasilissa splendida* genome, phylogenetic relationship, and gene family evolution. (**a**) *Himalopsyche anomala*. (**b**) *Eubasilissa splendida*. From the inner to outer layers: gene density, GC content (GC), DNA transposons (DNA), long-interspersed elements (LINE), long-terminal repeat elements (LTR), short-interspersed elements (SINE), chromosome length (Chr).

We integrated a multifaceted approach encompassing *ab initio* predictions, homologous proteins, and transcriptomic strategies to predict gene structures in the *H. anomala* and *E. splendida* genomes. Initially, we used BRAKER v2.1.6^38^, which integrated results from Augustus v3.3.3^39^ and GeneMark v4.32^40^. In this process, we utilized the arthropod reference proteins from OrthoDB10 v10^41^ to proceed *ab initio* predictions. Additionally, we downloaded the protein sequences of model organisms and closely related species (Table 3), including *Drosophila melanogaster* Meigen, *Bombyx mori* (Linnaeus), *Spodoptera litura* (Fabricius) and so on. These sequences were used for homologous gene prediction, employing GeMoMa v1.7.1^42^ with the parameter “GeMoMa.c = 0.5 GeMoMa.p = 10”. Transcriptome sequencing reads underwent the same quality control methods used for DNA sequencing. Subsequently, HISAT2 v2.2.0^43^ and samtools were employed to produce BAM alignments for reference assembly, and StringTie v2.1.6^44^ was used to perform transcriptome assembly. Conclusively, we used MAKER v3.01.03^45^ to synthesize the three distinct strategies. A total of 11,469 and 10,554 PCGs were predicted in the *H. anomala* and *E. splendida* genomes, respectively (Table 4). The average number of exons and introns per gene was similar in *H. anomala* (9.4 exons and 8.2 introns) and *E. splendida* (7.1 exons and 8.3 introns). Variations in gene density were observed across different chromosomes, with the highest gene density on chromosome 21 and chromosome 23 in the *H. anomala* and *E. splendida* genomes, respectively (Fig. 2a,b). BUSCO was employed to predict protein sequence for both genomes with integrity of 98.4% in protein model, attesting to the high-quality annotation of the genomes.

**Table 3:** Species taxonomic information and accession code of all samples used in this study.

Species	Class	Order	Source
*Tribolium castaneum*	Insecta	Coleoptera	NCBI (GCF_000002335.3)
*Drosophila melanogaster*	Insecta	Diptera	NCBI (GCF_000001215.4)
*Bombyx mori*	Insecta	Lepidoptera	NCBI (GCF_014905235.1)
*Helicoverpa armigera*	Insecta	Lepidoptera	NCBI (GCF_023701775.1)
*Spodoptera litura*	Insecta	Lepidoptera	NCBI (GCF_002706865.1)
*Cheumatopsyche charites*	Insecta	Trichoptera	10.6084/m9.figshare.19673562.v1

**Table 4:** Structural annotation information of protein-encoding genes of *Himalopsyche anomala* and *Eubasilissa splendida*.

Structural annotation	*Himalopsyche anomala*	*Eubasilissa splendida*
Number of protein-coding genes	11,469	10,554
Number of predicted protein sequences	13,652	12,736
Mean protein length (aa)	576.5	576.4
Mean gene length (bp)	12,237.20	14,481.30
Gene ratio	21.15%	17.79%
Number of exons per gene	9.4	7.1
Mean exon length (bp)	347.3	330.8
Exon ratio	5.70%	3.88%
Number of CDSs per gene	9.2	9.3
Mean CDS length (bp)	223	223.9
CDS ratio	3.56%	2.57%
Number of introns per gene	8.2	8.3
Mean intron length (bp)	1,084.10	1,358.4
Intron ratio	15.45%	13.91%

To functionally annotate the PCGs, Diamond v2.0.11.149^46^ was applied to search against the UniProtKB database^47^, using a sensitive strategy. Furthermore, eggNOGmapper v2.0.1^48^ was used to annotate protein domains based on eggNOG v5.0^49^. Concurrently, InterProScan 5.53–87.0^50^ was also employed to identify domains by Pfam^51^, SMART^52^, Superfamily^53^, Gene3D^54^, and CDD^55^ databases. Integration of the predicted results led to the functional annotation of 10,715 (93.42%) and 9,947 (94.24%) PCGs for *H. anomala* and *E. splendida*, respectively (Table S7).

## Data Records

The newly assembled genomes are available at the NCBI under the BioProject IDs: PRJNA749930 (*H. anomala*) and PRJNA749861 (*E. splendida*). Raw Illumina, PacBio, Hi-C, and transcriptome data for both species have been deposited in the Sequence Read Archive under identification numbers SRP351561 (*H. anomala*)^56^ and SRP351440 (*E. splendida*)^57^. The chromosomal assemblies of *H. anomala* and *E. splendida* have been deposited in the NCBI assembly with the accession numbers JAHZMQ000000000^58^ and JAHZML000000000^59^, respectively. Results of annotation for repetitive elements and gene prediction for both species are available in the figshare database^60^.

## Technical Validation

We evaluated the quality of *H. anomala* and *E. splendida* genome assemblies, focusing on completeness and accuracy. The completeness of assembly was evaluated using BUSCO with the insects_odb10 database, yielding final assemblies with BUSCO completeness of 98.1% and 98.2% for *H. anomala* and *E. splendida*, respectively, affirming the high quality of these genomes. To verify accuracy of assembly, we calculated mapping rates by aligning PacBio and Illumina reads to the final assembly: for *H. anomala*, 96.21%, 96.99%, and 96.41% of reads were successfully mapped, respectively; for *E. splendida*, higher mapping rates of 96.99%, 97.11%, and 96.42% were obtained, respectively. The Hic assembly underwent manual correction to ensure accuracy, and the Hi-C heatmap showed a well-organized interaction pattern at the chromosomal level (Fig. 1). Additionally, the final annotated gene BUSCO completeness was 98.4% for both *H. anomala* and *E. splendida*. Collectively, these results confirm the high quality and accuracy of the new chromosome-level assemblies.

### Supplementary information

Supporting information

## Data Availability

No specific code was used in this study. All analytical processes were executed according to the manuals and protocols of the corresponding bioinformatic tools.
