# Supplementary material for: Chromosome-scale genome assemblies of Himalopsyche anomala and Eubasilissa splendida (Insecta: Trichoptera)
Source: Sci Data. 2024 Mar 5;11:267. doi: 10.1038/s41597-024-03097-3 (PMC10914795; doi:10.1038/s41597-024-03097-3)
Supplement: Supplementary file 1 — Supporting information [file 41597_2024_3097_MOESM1_ESM.pdf]

Supporting Information Content

Figure S1······ **page2**
Figure S2······ **page2**
Table S1······ **page3**
Table S2······ **page4**
Table S3······ **page5**
Table S4······ **page9**
Table S5······ **page14**
Table S6······ **page17**
Table S7······ **page20**

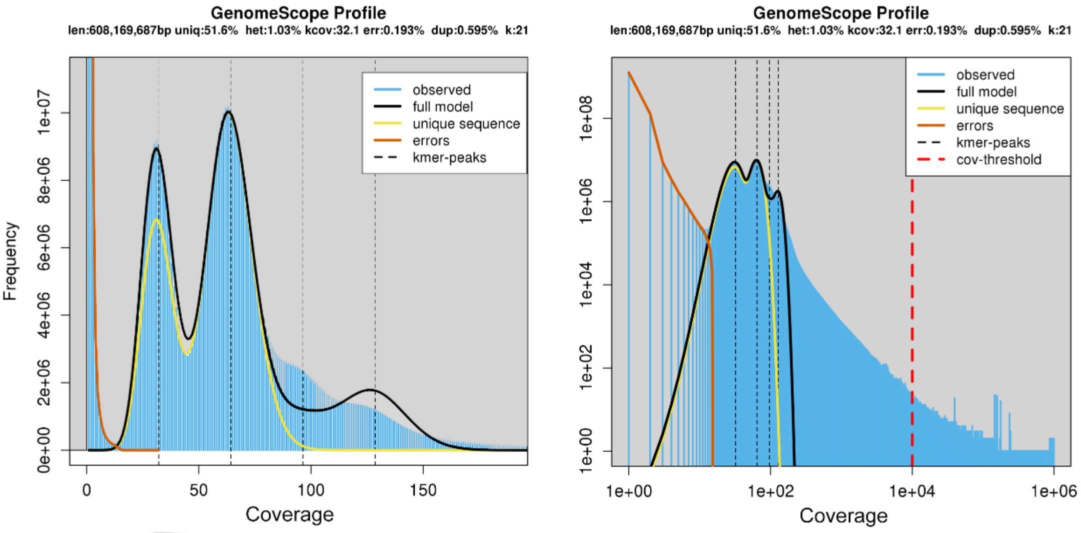

Figure S1. k-mers analysis of *Himalopsyche anomala* base on k=21

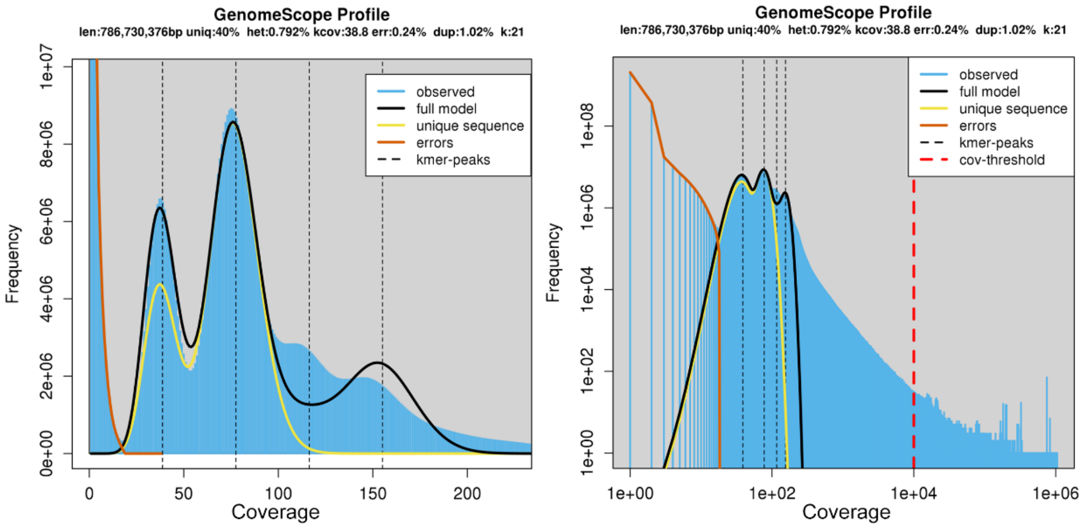

Figure S2. k-mers analysis of *Eubasilissa splendida* base on k=21

Table S1 The mapping rate of *Himalopsyche anomala* female illumina data in each chromosome.

**Chromosome number**	**Chromosome coverage rate**
Hano_Chr01	37.80
Hano_Chr02	37.77
Hano_Chr03	38.06
Hano_Chr04	37.67
Hano_Chr05	37.55
Hano_Chr06	39.00
Hano_Chr07	37.23
Hano_Chr08	37.42
Hano_Chr09	37.88
Hano_Chr10	37.20
Hano_Chr11	37.40
Hano_Chr12	37.11
Hano_Chr13	37.28
Hano_Chr14	37.06
Hano_Chr15	37.61
Hano_Chr16	37.06
Hano_Chr17	37.12
Hano_Chr18	37.26
Hano_Chr19	37.42
Hano_Chr20	37.13
Hano_Chr21	37.38
Hano_Chr22	38.17
Hano_Chr23	40.36
Hano_ChrZ	20.94

Table S2 The mapping rate of *Eubasilissa splendida* female illumina data in each chromosome.

Chromosome number	Chromosome coverage rate
Espl_Chr01	45.85
Espl_Chr02	44.95
Espl_Chr03	44.39
Espl_Chr04	44.53
Espl_Chr05	44.21
Espl_Chr06	45.06
Espl_Chr07	44.94
Espl_Chr08	44.22
Espl_Chr09	44.85
Espl_Chr10	44.70
Espl_Chr11	44.46
Espl_Chr12	44.47
Espl_Chr13	44.40
Espl_Chr14	44.36
Espl_Chr15	44.54
Espl_Chr16	45.44
Espl_Chr17	44.27
Espl_Chr18	44.18
Espl_Chr19	44.59
Espl_Chr20	46.93
Espl_Chr21	45.03
Espl_Chr22	44.18
Espl_Chr23	44.53
Espl_Chr24	44.57
Espl_Chr25	45.50
Espl_Chr26	44.57
Espl_Chr27	47.40
Espl_Chr28	46.66
Espl_ChrZ	24.22

Table S3 Statistics of the repetitive sequences identified in *Himalopsyche anomala*

Type	Number	Length (bp)	Percentage of genome
**DNA**	12,053	1,587,471	0.24%
Academ-1	92	4,922	0.00%
Academ-2	13	756	0.00%
Academ-H	5	288	0.00%
CMC	3	109	0.00%
CMC-Chapaev	113	5,612	0.00%
CMC-Chapaev-3	58	9,900	0.00%
CMC-EnSpm	13,373	1,586,767	0.24%
CMC-Transib	122	6,809	0.00%
Crypton	21	756	0.00%
Crypton-A	20	974	0.00%
Crypton-C	2	147	0.00%
Crypton-F	8	331	0.00%
Crypton-H	60	2,641	0.00%
Crypton-I	4	208	0.00%
Crypton-R	1	42	0.00%
Crypton-S	11	602	0.00%
Crypton-V	556	46,303	0.01%
Crypton-X	3	145	0.00%
Dada	90	3,863	0.00%
Ginger	5	333	0.00%
Ginger-1	63	2,840	0.00%
Ginger-2	73	10,431	0.00%
IS3EU	51	2,722	0.00%
Kolobok	9	497	0.00%
Kolobok-E	524	277,536	0.04%
Kolobok-H	10	502	0.00%
Kolobok-Hydra	688	169,729	0.03%
Kolobok-T2	125	5,797	0.00%
MULE	4	281	0.00%
MULE-F	4	201	0.00%
MULE-MuDR	6,270	498,243	0.08%
MULE-NOF	61	3,650	0.00%
MULE-Ricksha	4	159	0.00%
Maverick	533	82,288	0.01%
Maverick-Mavirus	5	295	0.00%
Merlin	590	122,466	0.02%
Novosib	5	525	0.00%
P	467	27,926	0.00%
P-Fungi	14	723	0.00%
PIF-HarbS	6	314	0.00%
PIF-Harbinger	808	184,142	0.03%

PIF-ISL2EU	114	8,599	0.00%
PIF-Spy	46	2,585	0.00%
PiggyBac	2,844	1,542,477	0.23%
PiggyBac-A	1	111	0.00%
PiggyBac-X	20	1,034	0.00%
Sola-1	6,448	1,356,582	0.20%
Sola-2	90	5,191	0.00%
Sola-3	41	2,540	0.00%
TcMar	315	23,493	0.00%
TcMar-Ant1	4	224	0.00%
TcMar-Cweed	4	181	0.00%
TcMar-Fot1	164	9,802	0.00%
TcMar-Gizmo	1	49	0.00%
TcMar-IS885	4	161	0.00%
TcMar-ISRm11	8,115	1,002,957	0.15%
TcMar-Mariner	41,004	8,356,073	1.26%
TcMar-Mogwai	4	283	0.00%
TcMar-Pogo	813	292,824	0.04%
TcMar-Sagan	14	708	0.00%
TcMar-Stowaway	30	1,340	0.00%
TcMar-Tc1	5,477	1,073,893	0.16%
TcMar-Tc2	1,953	306,195	0.05%
TcMar-Tc4	10,407	934,179	0.14%
TcMar-Tigger	57,511	11,239,254	1.69%
TcMar-m44	34	4,609	0.00%
Zator	434	40,242	0.01%
Zisupton	126	6,336	0.00%
hAT	6,179	890,569	0.13%
hAT-Ac	7,920	902,915	0.14%
hAT-Blackjack	3,965	416,295	0.06%
hAT-Charlie	142,171	20,057,252	3.02%
hAT-Pegasus	144	19,910	0.00%
hAT-Restless	1	41	0.00%
hAT-Tag1	100	4,973	0.00%
hAT-Tip100	113,995	16,624,259	2.51%
hAT-hAT1	2	136	0.00%
hAT-hAT19	1,026	186,370	0.03%
hAT-hAT5	11	520	0.00%
hAT-hATm	131	7,129	0.00%
hAT-hATw	13	689	0.00%
hAT-hATx	112,637	15,924,223	2.40%
hAT-hobo	250	18,112	0.00%
**LINE**	311	37,071	0.01%
CR1	8,727	2,701,845	0.41%

CR1-Zenon	32	1,530	0.00%
CRE	85	11,510	0.00%
CRE-Ambal	3	119	0.00%
CRE-Odin	17	888	0.00%
Deceiver	3	162	0.00%
Dong-R4	21	1,141	0.00%
Dualen	3	172	0.00%
I	214	12,172	0.00%
I-Jockey	9,117	3,522,622	0.53%
L1	779	41,067	0.01%
L1-DRE	3	111	0.00%
L1-Tx1	4,833	275,324	0.04%
L1-Zorro	1	81	0.00%
L2	906	64,271	0.01%
Penelope	70,569	9,077,282	1.37%
Proto1	3	179	0.00%
Proto2	20	951	0.00%
R1	9,762	2,508,801	0.38%
R1-LOA	37	2,027	0.00%
R2	58	3,290	0.00%
R2-Hero	9	526	0.00%
R2-NeSL	42	2,856	0.00%
RTE	102	9,808	0.00%
RTE-BovB	38,786	6,832,134	1.03%
RTE-ORTE	3	193	0.00%
RTE-RTE	35	1,581	0.00%
RTE-X	91	5,094	0.00%
Rex-Babar	25	1,050	0.00%
Tad1	66	3,323	0.00%
**LTR**	56	4,370	0.00%
Caulimovirus	199	11,700	0.00%
Copia	1,941	106,370	0.02%
DIRS	200	60,287	0.01%
DIRS-Q	4	228	0.00%
ERV	1	39	0.00%
ERV-Foamy	4	216	0.00%
ERV-Lenti	1	72	0.00%
ERV1	377	18,286	0.00%
ERV4	26	1,365	0.00%
ERVK	193	9,855	0.00%
ERVL	33	1,375	0.00%
ERVL-MaLR	2	58	0.00%
Gypsy	7,615	1,301,620	0.20%
Ngaro	39	2,597	0.00%

Pao	604	34,457	0.01%
**DNA_virus**	4	260	0.00%
**RC**	--	--	--
Helitron	50,786	14,305,080	2.16%
Helitron-2	5	269	0.00%
**Retroposon**	7	410	0.00%
L1-dep	1	115	0.00%
SVA	1	80	0.00%
**SINE**	6	343	0.00%
5S-Deu-L2	1	56	0.00%
5S-RTE	1	50	0.00%
B2	1	59	0.00%
ID	7	288	0.00%
MIR	8,870	636,146	0.10%
tRNA	26,079	4,050,312	0.61%
tRNA-7SL	1	98	0.00%
tRNA-CR1	2	180	0.00%
tRNA-Core	1,634	171,569	0.03%
tRNA-Deu-L2	2	123	0.00%
tRNA-Deu-RTE	1	21	0.00%
tRNA-L2	4	298	0.00%
tRNA-Meta	12,869	3,927,529	0.59%
tRNA-RTE	17,804	3,201,739	0.48%
tRNA-Rex	1	20	0.00%
tRNA-V	4	47	0.00%
tRNA-V-CR1	1	74	0.00%
Unknown	989,173	142,138,755	21.43%
centromeric	1	58	0.00%
Unspecified	1	68	0.00%
Total_interspersed	1,824,717	281,021,779	42.36%
Low_complexity	19,139	954,907	0.14%
Satellite	1,037	178,784	0.03%
Y-chromosome	1	75	0.00%
centr	2	97	0.00%
macro	4	165	0.00%
Simple_repeat	87,667	4,689,010	0.71%
rRNA	695	89,209	0.01%
scRNA	1	60	0.00%
snRNA	45	5,912	0.00%
srpRNA	4	1,184	0.00%
tRNA	7,898	1,158,907	0.17%
Total	1,941,210	288,100,089	43.43%

Table S4 Statistics of the repetitive sequences identified in *Eubasilissa splendida*

Type	Number	Length (bp)	Percentage of genome
**DNA**	7,206	1,333,338	0.16%
Academ-1	463	73,720	0.01%
Academ-2	8	384	0.00%
Academ-H	7	446	0.00%
CMC	2	110	0.00%
CMC-Chapaev	265	11,838	0.00%
CMC-Chapaev-3	4,178	1,289,959	0.15%
CMC-EnSpm	4,768	355,750	0.04%
CMC-Mirage	6	393	0.00%
CMC-Transib	178	16,211	0.00%
Crypton	70	2,422	0.00%
Crypton-A	9	461	0.00%
Crypton-C	7	456	0.00%
Crypton-F	7	416	0.00%
Crypton-H	1,129	191,239	0.02%
Crypton-I	1	58	0.00%
Crypton-S	19	994	0.00%
Crypton-V	468	14,311	0.00%
Crypton-X	3	160	0.00%
Dada	237	11,182	0.00%
Ginger-1	189	10,977	0.00%
Ginger-2	171	52,983	0.01%
IS3EU	913	38,655	0.00%
Kolobok	19	1,070	0.00%
Kolobok-E	326	69,671	0.01%
Kolobok-H	7	435	0.00%
Kolobok-Hydra	634	157,433	0.02%
Kolobok-T2	369	15,775	0.00%
MULE	2	98	0.00%
MULE-F	6	302	0.00%
MULE-MuDR	2,970	367,094	0.04%
MULE-NOF	65	3,540	0.00%
MULE-Ricksha	3	380	0.00%
Maverick	752	41,320	0.00%
Maverick-Mavirus	5	244	0.00%
Merlin	136	8,450	0.00%
Novosib	33	2,375	0.00%
P	1,988	377,536	0.04%
P-Fungi	11	375	0.00%
PIF	2	126	0.00%
PIF-HarbS	6	295	0.00%
PIF-Harbinger	4,563	1,677,594	0.20%

PIF-ISL2EU	66	3,587	0.00%
PIF-Spy	106	5,416	0.00%
PiggyBac	4,526	1,998,493	0.23%
PiggyBac-A	1	75	0.00%
PiggyBac-X	16	809	0.00%
Sola-1	8,376	1,895,822	0.22%
Sola-2	1,783	377,273	0.04%
Sola-3	70	4,552	0.00%
TcMar	71	3,153	0.00%
TcMar-Ant1	8	424	0.00%
TcMar-Cweed	5	320	0.00%
TcMar-Fot1	4,302	931,189	0.11%
TcMar-Gizmo	1	49	0.00%
TcMar-IS885	3	137	0.00%
TcMar-ISRm11	101	6,025	0.00%
TcMar-Mariner	55,903	14,426,334	1.68%
TcMar-Mogwai	1	33	0.00%
TcMar-Pogo	2,716	1,208,414	0.14%
TcMar-Sagan	9	408	0.00%
TcMar-Stowaway	35	1,463	0.00%
TcMar-Tc1	95,705	26,308,538	3.06%
TcMar-Tc2	4,748	944,429	0.11%
TcMar-Tc4	76	4,071	0.00%
TcMar-Tigger	50,326	10,602,434	1.23%
TcMar-m44	234	45,093	0.01%
Zator	276	89,402	0.01%
Zisupton	486	21,076	0.00%
hAT	3,373	653,475	0.08%
hAT-Ac	20,918	2,051,972	0.24%
hAT-Blackjack	1,920	426,651	0.05%
hAT-Charlie	36,560	4,755,719	0.55%
hAT-Pegasus	18	822	0.00%
hAT-Restless	1	171	0.00%
hAT-Tag1	216	10,230	0.00%
hAT-Tip100	20,966	3,285,769	0.38%
hAT-hAT19	34	2,167	0.00%
hAT-hAT5	16	749	0.00%
hAT-hAT6	3	202	0.00%
hAT-hATm	1,216	332,811	0.04%
hAT-hATw	18	1,081	0.00%
hAT-hATx	9,559	1,684,170	0.20%
hAT-hobo	58	3,908	0.00%
**LINE**	1,078	128,350	0.01%
CR1	31,049	6,730,212	0.78%

CR1-Zenon	218	16,562	0.00%
CRE	25	1,138	0.00%
CRE-Ambal	4	177	0.00%
CRE-Odin	32	1,541	0.00%
Deceiver	11	526	0.00%
Dong-R4	25	1,471	0.00%
Dualen	3	70	0.00%
I	19,637	5,018,795	0.58%
I-Jockey	9,259	2,698,195	0.31%
L1	2,511	397,446	0.05%
L1-DRE	6	287	0.00%
L1-Tx1	291	14,944	0.00%
L1-Zorro	1	29	0.00%
L2	121,726	25,389,715	2.95%
Penelope	51,067	10,367,422	1.21%
Proto1	3	134	0.00%
Proto2	11,011	2,696,875	0.31%
R1	4,723	1,014,033	0.12%
R1-LOA	302	115,992	0.01%
R2	180	44,953	0.01%
R2-Hero	13	671	0.00%
R2-NeSL	49	3,522	0.00%
RTE	2,030	230,295	0.03%
RTE-BovB	266,278	48,545,670	5.65%
RTE-ORTE	6	296	0.00%
RTE-RTE	33,779	4,486,136	0.52%
RTE-X	209	10,479	0.00%
Rex-Babar	838	153,959	0.02%
Tad1	79	4,038	0.00%
**LTR**	126	7,632	0.00%
Caulimovirus	172	9,620	0.00%
Copia	2,246	141,793	0.02%
DIRS	616	193,608	0.02%
DIRS-Q	5	252	0.00%
ERV	1	42	0.00%
ERV-Foamy	5	386	0.00%
ERV-Lenti	1	59	0.00%
ERV1	1,905	330,298	0.04%
ERV4	34	2,004	0.00%
ERVK	299	16,016	0.00%
ERVL	64	2,582	0.00%
ERVL-MaLR	6	404	0.00%
Gypsy	13,420	1,855,387	0.22%
Ngaro	123	7,482	0.00%

Pao	1,695	1,230,463	0.14%
Viper	1	59	0.00%
**DNA_virus**	4	200	0.00%
**RC**	--	--	--
Helitron	14,882	2,772,937	0.32%
Helitron-2	11	422	0.00%
**Retroposon**	2	37	0.00%
**SINE**	85,349	12,999,365	1.51%
5S-Deu	2	68	0.00%
5S-Sauria-RTE	2	45	0.00%
B2	1	36	0.00%
B4	2	59	0.00%
ID	28	1,104	0.00%
MIR	166	3,951	0.00%
tRNA	3,257	184,459	0.02%
tRNA-Ceph-RTE	11	223	0.00%
tRNA-Core	9	288	0.00%
tRNA-Core-RTE	2	135	0.00%
tRNA-Deu	1	18	0.00%
tRNA-Deu-I	152	5,843	0.00%
tRNA-Deu-L2	2	42	0.00%
tRNA-Deu-RTE	3	108	0.00%
tRNA-I	3	162	0.00%
tRNA-L2	5	89	0.00%
tRNA-Meta	4,810	332,104	0.04%
tRNA-RTE	13	495	0.00%
tRNA-V	18	830	0.00%
tRNA-V-CR1	1	20	0.00%
**Unknown**	1,558,235	244,380,176	28.44%
centromeric	3	176	0.00%
Total_interspersed	2,601,165	450,775,020	52.46%
Low_complexity	27,290	1,288,155	0.15%
Satellite	13,636	1,067,107	0.12%
W-chromosome	1	58	0.00%
Y-chromosome	3	177	0.00%
acro	4	123	0.00%
centr	3	693	0.00%
macro	10	216	0.00%
subtelo	1	42	0.00%
Simple_repeat	159,017	7,313,718	0.85%
rRNA	200	39,802	0.00%
scRNA	1	11	0.00%
snRNA	61	7,051	0.00%
srpRNA	2	572	0.00%

tRNA	92,010	10,733,095	1.25%
Total	2,893,404	471,225,840	54.84%

Table S5 Statistics of the ncRNA sequences annotated in *Himalopsyche anomala*

Class	Copies	Description	
**rRNA**			
5.8S_rRNA	2	rRNA	5.8S ribosomal RNA
5S_rRNA	114	rRNA	5S ribosomal RNA
LSU_rRNA_eukarya	6	rRNA	Eukaryotic large subunit ribosomal RNA
SSU_rRNA_eukarya	2	rRNA	Eukaryotic small subunit ribosomal RNA
**miRNA**			
bantam	1	miRNA	microRNA bantam
let-7	1	miRNA	let-7 microRNA precursor
mir-1	1	miRNA	mir-1 microRNA precursor family
mir-10	3	miRNA	mir-10 microRNA precursor family
mir-1000	1	miRNA	mir-1000 microRNA precursor family
mir-11	1	miRNA	mir-11 microRNA precursor family
mir-1175	1	miRNA	mir-1175 microRNA precursor family
mir-124	1	miRNA	mir-124 microRNA precursor family
mir-133	1	miRNA	mir-133 microRNA precursor family
mir-137	1	miRNA	microRNA mir-137
mir-184	1	miRNA	microRNA mir-184
mir-190	1	miRNA	microRNA mir-190
mir-2	4	miRNA	mir-2 microRNA precursor
mir-210	1	miRNA	microRNA mir-210
mir-219	2	miRNA	mir-219 microRNA precursor family
mir-252	1	miRNA	microRNA mir-252
mir-263	2	miRNA	microRNA mir-263
mir-275	1	miRNA	microRNA mir-275
mir-2755	1	miRNA	mir-2755 microRNA precursor family
mir-276	1	miRNA	microRNA mir-276
mir-2765	1	miRNA	mir-2765 microRNA precursor family
mir-277	1	miRNA	microRNA mir-277
mir-2788	1	miRNA	mir-2788 microRNA precursor family
mir-2796	1	miRNA	mir-2796 microRNA precursor family
mir-282	1	miRNA	microRNA mir-282
mir-283	1	miRNA	microRNA mir-283
mir-305	1	miRNA	microRNA mir-305
mir-31	1	miRNA	microRNA mir-31
mir-317	1	miRNA	microRNA mir-317
mir-449	1	miRNA	microRNA mir-449
mir-46	1	miRNA	mir-46/mir-47/mir-281 microRNA precursor family
mir-67	1	miRNA	microRNA mir-67
mir-7	1	miRNA	mir-7 microRNA precursor

mir-71	1	miRNA	microRNA mir-71
mir-8	2	miRNA	mir-8/mir-141/mir-200 microRNA precursor family
MIR821	6	miRNA	microRNA MIR821
mir-9	3	miRNA	mir-9/mir-79 microRNA precursor family
mir-927	1	miRNA	microRNA mir-927
mir-929	1	miRNA	microRNA mir-929
mir-932	1	miRNA	microRNA mir-932
mir-981	1	miRNA	microRNA mir-981
mir-iab-4	1	miRNA	mir-iab-4 microRNA precursor family
**snRNA**			
SNORA53	1	snRNA; snoRNA; HACA-box	Small nucleolar RNA SNORA53
SNORD31	3	snRNA; snoRNA; CD-box	Small nucleolar RNA SNORD31
SNORD36	1	snRNA; snoRNA; CD-box	Small nucleolar RNA SNORD36
SNORD49	1	snRNA; snoRNA; CD-box	Small nucleolar RNA SNORD49
snosnR60_Z15	4	snRNA; snoRNA; CD-box	Small nucleolar RNA snR60/Z15/Z230/Z193/J17
snosnR61	1	snRNA; snoRNA; CD-box	Small nucleolar RNA snR61/Z1/Z11
snoU13	1	snRNA; snoRNA; CD-box	Small nucleolar RNA U13
U1	15	snRNA; splicing	U1 spliceosomal RNA
U11	2	snRNA; splicing	U11 spliceosomal RNA
U12	1	snRNA; splicing	U12 minor spliceosomal RNA
U2	11	snRNA; splicing	U2 spliceosomal RNA
U3	7	snRNA; snoRNA; CD-box	Small nucleolar RNA U3
U4	2	snRNA; splicing	U4 spliceosomal RNA
U4atac	1	snRNA; splicing	U4atac minor spliceosomal RNA
U5	9	snRNA; splicing	U5 spliceosomal RNA
U6	26	snRNA; splicing	U6 spliceosomal RNA
U6atac	2	snRNA; splicing	U6atac minor spliceosomal RNA
**lncRNA**			
Sphinx_1	1	lncRNA	Sphinx_ conserved region 1
Sphinx_2	1	lncRNA	Sphinx conserved region 2
**ribozyme**			
Hammerhead_3	2	ribozyme	Hammerhead ribozyme (type III)
RNase_MRP	1	ribozyme	RNase MRP
RNaseP_nuc	2	ribozyme	Nuclear RNase P

**tRNA**		
tRNA-Ala	29	tRNA
tRNA-Arg	26	tRNA
tRNA-Asn	19	tRNA
tRNA-Asp	19	tRNA
tRNA-Cys	11	tRNA
tRNA-Gln	16	tRNA
tRNA-Glu	27	tRNA
tRNA-Gly	39	tRNA
tRNA-His	12	tRNA
tRNA-Ile	12	tRNA
tRNA-iMet	15	tRNA
tRNA-Leu	26	tRNA
tRNA-Lys	21	tRNA
tRNA-Met	8	tRNA
tRNA-Phe	12	tRNA
tRNA-Pro	26	tRNA
tRNA-Ser	25	tRNA
tRNA-Thr	17	tRNA
tRNA-Trp	10	tRNA
tRNA-Tyr	14	tRNA
**Others**		
Arthropod_7SK	1	Arthropod 7SK RNA
Histone3	47	Histone 3' UTR stem-loop
IRE_II	1	Iron response element II
K_chan_RES	4	Potassium channel RNA editing signal
Metazoa_SRP	4	Metazoan signal recognition particle RNA
Total	741	

Table S6 Statistics of the ncRNA sequences annotated in *Eubasilissa splendida*

**Class**	**Copies**	**Description**	
**rRNA**			
5.8S_rRNA	5	rRNA	5.8S ribosomal RNA
5S_rRNA	92	rRNA	5S ribosomal RNA
LSU_rRNA_archaea	1	rRNA	Archaeal large subunit ribosomal RNA
LSU_rRNA_eukarya	16	rRNA	Eukaryotic large subunit ribosomal RNA
SSU_rRNA_eukarya	7	rRNA	Eukaryotic small subunit ribosomal RNA
**miRNA**			
bantam	1	miRNA	microRNA bantam
let-7	1	miRNA	let-7 microRNA precursor
mir-1	1	miRNA	mir-1 microRNA precursor family
mir-10	3	miRNA	mir-10 microRNA precursor family
mir-1000	1	miRNA	mir-1000 microRNA precursor family
mir-11	1	miRNA	mir-11 microRNA precursor family
mir-1175	1	miRNA	mir-1175 microRNA precursor family
mir-124	1	miRNA	mir-124 microRNA precursor family
mir-133	1	miRNA	mir-133 microRNA precursor family
mir-137	1	miRNA	microRNA mir-137
mir-184	1	miRNA	microRNA mir-184
mir-190	1	miRNA	microRNA mir-190
mir-2	5	miRNA	mir-2 microRNA precursor
mir-210	1	miRNA	microRNA mir-210
mir-219	2	miRNA	mir-219 microRNA precursor family
mir-252	1	miRNA	microRNA mir-252
mir-263	2	miRNA	microRNA mir-263
mir-276	1	miRNA	microRNA mir-276
mir-2765	1	miRNA	mir-2765 microRNA precursor family
mir-277	1	miRNA	microRNA mir-277
mir-2788	1	miRNA	mir-2788 microRNA precursor family
mir-2796	1	miRNA	mir-2796 microRNA precursor family
mir-282	1	miRNA	microRNA mir-282
mir-305	1	miRNA	microRNA mir-305
mir-31	1	miRNA	microRNA mir-31
mir-317	1	miRNA	microRNA mir-317
mir-33	1	miRNA	microRNA mir-33
mir-449	1	miRNA	microRNA mir-449
mir-46	1	miRNA	mir-46/mir-47/mir-281 microRNA precursor family
mir-67	1	miRNA	microRNA mir-67
mir-71	1	miRNA	microRNA mir-71

mir-8	2	miRNA	mir-8/mir-141/mir-200 microRNA precursor family
mir-9	3	miRNA	mir-9/mir-79 microRNA precursor family
mir-927	1	miRNA	microRNA mir-927
mir-929	1	miRNA	microRNA mir-929
mir-932	1	miRNA	microRNA mir-932
mir-965	1	miRNA	mir-965 microRNA precursor family
mir-971	1	miRNA	mir-971 microRNA precursor family
mir-981	1	miRNA	microRNA mir-981
mir-iab-4	1	miRNA	mir-iab-4 microRNA precursor family
**snRNA**			
snoMe28S-Am982	1	snRNA; snoRNA; CD-box	Small nucleolar RNA Me28S-Am982
SNORD31	3	snRNA; snoRNA; CD-box	Small nucleolar RNA SNORD31
SNORD36	3	snRNA; snoRNA; CD-box	Small nucleolar RNA SNORD36
snosnR60_Z15	4	snRNA; snoRNA; CD-box	Small nucleolar RNA snR60/Z15/Z230/Z193/J17
snosnR61	1	snRNA; snoRNA; CD-box	Small nucleolar RNA snR61/Z1/Z11
U1	15	snRNA; splicing	U1 spliceosomal RNA
U11	1	snRNA; splicing	U11 spliceosomal RNA
U12	1	snRNA; splicing	U12 minor spliceosomal RNA
U2	10	snRNA; splicing	U2 spliceosomal RNA
U3	8	snRNA; snoRNA; CD-box	Small nucleolar RNA U3
U4	4	snRNA; splicing	U4 spliceosomal RNA
U4atac	1	snRNA; splicing	U4atac minor spliceosomal RNA
U5	9	snRNA; splicing	U5 spliceosomal RNA
U6	9	snRNA; splicing	U6 spliceosomal RNA
U6atac	1	snRNA; splicing	U6atac minor spliceosomal RNA
**lncRNA**			
Sphinx_2	1	lncRNA	Sphinx conserved region 2
**ribozyme**			
Hammerhead_3	1	ribozyme	Hammerhead ribozyme (type III)
RNase_MRP	1	ribozyme	RNase MRP
RNaseP_nuc	2	ribozyme	Nuclear RNase P
**tRNA**			
tRNA-Ala	32	tRNA	
tRNA-Arg	28	tRNA	
tRNA-Asn	19	tRNA	
tRNA-Asp	21	tRNA	

tRNA-Cys	6	tRNA
tRNA-Gln	11	tRNA
tRNA-Glu	25	tRNA
tRNA-Gly	35	tRNA
tRNA-His	12	tRNA
tRNA-Ile	15	tRNA
tRNA-iMet	13	tRNA
tRNA-Leu	28	tRNA
tRNA-Lys	25	tRNA
tRNA-Met	7	tRNA
tRNA-Phe	9	tRNA
tRNA-Pro	29	tRNA
tRNA-Ser	32	tRNA
tRNA-Thr	25	tRNA
tRNA-Trp	10	tRNA
tRNA-Tyr	13	tRNA
tRNA-Val	25	tRNA
**Others**		
Arthropod_7SK	1	Arthropod 7SK RNA
Histone3	91	Histone 3' UTR stem-loop
K_chan_RES	4	Potassium channel RNA editing signal
Metazoa_SRP	2	Metazoan signal recognition particle RNA
**Total**	766	

Table S7 Functional annotation information of protein-encoding genes of two trichopteran genome

**Functional annotation**	***Himalopsyche anomala***	***Eubasilissa splendida***
Number of genes with InterProScan annotations	9,687	10,371
Number of genes with GO items from InterProScan annotations	6,872	7,326
Number of genes with KEGG pathway items from InterProScan annotations	6,321	6,683
Number of genes with MetaCyc items from InterProScan annotations	6,541	6,929
Number of genes with Reactome items from InterProScan annotations	8,345	8,856
Number of genes matching Uniprot records	9,947	10,715
Number of genes labelled as "Uncharacterized protein"	552	701
Number of genes labelled as "unknown function"	639	797
Number of genes with GO items from eggNOG annotations	9,844	8,118
Number of genes with gene names (function) from eggNOG annotations	6,256	6,945
Number of genes with Enzyme Codes (EC) from eggNOG annotations	2,277	2,455
Number of genes with KEGG ko terms from eggNOG annotations	6,520	7,070
Number of genes with KEGG pathway terms from eggNOG annotations	3,933	4,341
Number of genes with COG Functional Categories from eggNOG annotations	9,391	10,095
Number of genes with GO items (combining InterProScan and eggNOG results)	8,405	9,404
Number of genes with KEGG pathways items (combining InterProScan and eggNOG results)	7,333	7,877
